# Focusing on ADHD: Analyzing Five-Year Search Trends

**DOI:** 10.1192/j.eurpsy.2025.1166

**Published:** 2025-08-26

**Authors:** C. Noureddine, A. Sterchele, K. Petersen

**Affiliations:** 1 Psychiatry, Icahn School of Medicine at Mount Sinai, New York, United States

## Abstract

**Introduction:**

An estimated 10% of children in the United States carry a diagnosis of ADHD (Li *et al.* JAMA Netw Open 2023; 6). Parents, caregivers, and children themselves utilize search engines to better understand their diagnosis and treatment options as well as discover other resources such as finding providers or support groups. Furthermore, evaluating search trends may elucidate individual and societal barriers towards accessing treatment. (Zhao *et al.* Adm Policy Ment Health 2022*;* ***49*** 357–373)

**Objectives:**

This study examines Google search trends for the term “ADHD” from 2019 to 2023 to shed light on public interest and awareness patterns.

**Methods:**

A Google Trends search was performed for the term “ADHD” in the global search database from 2019 to 2023. Weekly absolute search volumes were exported for each year. A one-way ANOVA calculation including Tukey HSD was conducted using Social Science Statistics’ calculator, which was cross referenced with AAT Bioquest’s calculator.

**Results:**

The ANOVA analysis revealed significant differences in the number of searches across the five different years. The f-ratio value is 689.19733. The p-value is < .00001. Post hoc comparisons using the Tukey HSD test indicated that search results differed significantly from among all years except between the years 2019 and 2020. The average number of searches steadily increased from M2019 = 278201.4839, SD2019=38184.594 and M2023 = 642020.8824, SD2023 =33099.9021. There is a relatively steady level of interest in the search term throughout the year with slight fluctuations. Seasonal trends were examined, showing an increased number of searches in February, October, and June, and decreased searches in December.

**Conclusions:**

Both the overall and temporal trends described in this study have important clinical ramifications. The general increase in search trend frequency suggests increased awareness not due to a significant change in prevalence. The seasonal trends noted above suggest that the increased search frequency is related to school-related activities, particularly year-end exams in June, while the decrease in December may reflect the shift in focus from academics during the holiday break. This study highlights the importance of having evidence-based resources be accessible and understandable to the general public

**Disclosure of Interest:**

None Declared

